# Supplementary material for: Are carbon emissions trading and green financial instruments synergistic? -Comprehensive quantitative research based on content analysis
Source: PLoS One. 2024 Mar 7;19(3):e0298601. doi: 10.1371/journal.pone.0298601 (PMC10919601; doi:10.1371/journal.pone.0298601)
Supplement: S1 Data — (ZIP) [file pone.0298601.s001.zip › Data/地级市Z_F工作报告.zip!/╕╩╦α/╜≡▓2╩╨/└┤╫╘╣1⁄2╓┌║┼í╛┬φ┐╦╩2╛▌═°í┐_2019.docx]

各位代表：

现在，我代表市人民政府，向大会作工作报告，请予审议，并请各位政协委员和列席会议的同志提出意见。

一、2018年工作回顾

2018年，在市委的坚强领导下，在市人大、市政协的监督支持下，我们坚持以习近平新时代中国特色社会主义思想为指导，深入学习贯彻党的十九大精神，全面落实习近平总书记视察甘肃重要讲话和“八个着力”重要指示精神，坚持稳中求进工作总基调，以新发展理念为引领，落实高质量发展要求，扎实推进“三大攻坚战”，着力推进稳增长、促改革、调结构、惠民生、防风险，全市经济稳步增长，产业布局持续优化，生态环境日益改善，民生保障不断加强，社会大局和谐稳定，较好完成八届人大二次会议确定的各项目标任务，朝着实现全面建成小康社会的目标迈出了新的步伐。

——转型发展开创新局面。坚持以供给侧结构性改革为主线，认真落实省上“三重”“三一”工作方案，制定实施促进经济稳定增长等一系列政策措施，把“十大生态产业”发展作为转方式调结构、发展实体经济、促进动能转换、建设现代化经济体系、推动高质量发展的结合点和总抓手，着力推动绿色转型升级，全市103户规模以上工业企业中有81户分属“十大生态产业”。预计实现地区生产总值263.3亿元，同比增长8.7%；规模以上工业增加值102.8亿元，增长12%；固定资产投资增长11.5%；一般公共预算收入23.08亿元，增长3.9%；社会消费品零售总额95.2亿元，增长7.5%；城镇和农村居民人均可支配收入分别达到37272元、14354元，增长7.5%和8%；居民消费价格指数上涨1.8%；单位GDP能耗下降0.5%以上；全市经济基本面更加稳固，向好势头更加明显，发展质量更加趋优。

——项目建设取得新成效。全面落实领导包抓、清单管理、联审联批、在线审批监管、招商引资项目交办流程和社会投资项目服务工作规则等制度，90个重大项目开复工率达92.2%，较上年提高2.7个百分点；实施招商引资项目192项，到位资金101.85亿元；第二十四届“兰洽会”签约项目25个，签约资金80.6亿元。供热管网新改建、农村公路新改建和养护维修及生命防护工程等项目有序开展，G570金昌（下四分）至永昌段、S301线破城子至军马四场段和S308线何家湾至永昌段改建、武威路至河雅路主车道大修、城市危桥加固改造等项目建成通车。新版城市总体规划纲要、大数据产业园控制性详细规划、街区路网结构优化实施方案、新三年棚户区改造计划编制完成，开工建设棚户区改造项目29个24695套。

——“工业强市”跃上新台阶。新时代地企融合深度发展，合作机制更加完善，合作领域更加广泛，合作成果日益显现，一批支持金川集团公司整体上市遗留问题有效解决。甘肃省镍钴新材料军民融合协同创新中心挂牌运行，3000吨高电压锂离子电池用四氧化三钴、3.5万吨硫酸镍扩能技术改造、2万吨高端耐磨铸件二期和3000吨有色金属功能性超微粉体材料、含铜冶炼废渣综合利用、6万吨岩棉部分生产线等项目建成投运，“紫金云”大数据产业园数据中心一期、3万吨动力锂离子电池用三元前驱体、3.5万吨高端电镀用镍盐等项目有序推进，规模以上企业效益大幅提升，预计利润同比增长2.1倍、利税增长20%。列支“工业强市”专项资金4500万元，支持氯碱化工产业链降低用电成本、重点企业产能释放、小微企业升规入统，氯碱化工循环产业链生产负荷达到80%，鑫华焦化2号炉顺利启运。中小企业贷款余额112亿元，发放“助保贷”“互助贷”余额2.3亿元，为中小企业降低融资成本900万元。培育国家高新技术企业9家、省级以上知识产权优势企业3家、省级科技创新型企业6家，金川公司1万吨羰基镍生产线投料试生产，进入完全拥有自主知识产权的世界羰化冶金先进行列。

——乡村振兴迈出新步伐。坚持把脱贫攻坚作为首要政治责任和第一民生工程，全面夯实精准帮扶、产业扶贫、各方责任、基层队伍、工作作风“五个基础”，建立完善领导干部包抓建档立卡贫困户脱贫责任制，统筹财政专项扶贫资金7101万元，精准实施到村到户扶贫项目，毛家庄村、邵家庄村、马营沟村整体脱贫，全市250户703人实现脱贫。乡村振兴战略实施规划编制完成，新时代全省城乡融合发展示范区建设步伐加快，农村公共基础设施共管共享工作全面展开，粮食生产功能区划定工作基本完成，高原夏菜、优质肉羊、现代制种、食用菌等现代特色优势产业稳步发展，新改建标准化养殖小区12个，建成蔬菜绿色基地5个，蔬菜种植面积达到28万亩，戈壁农业面积达到2335亩，胡萝卜、大麦茶、藜麦、“盐滩青”萝卜等“金”字号农产品市场影响力和竞争力不断扩大，热销香港、深圳、广州、杭州、南京等地，外销量达80万吨。国家农产品质量安全市创建工作稳步推进，重大动物疫病防治工作进一步加强，推广农业新品种新技术6.9万亩、高效节水灌溉10.1万亩，“三品一标”农产品达65%以上，农畜产品抽检合格率达99.9%。农村一二三产业深度融合，培育省级农业科技示范园区2个，新认定市级新型农业经营主体42家、省级龙头企业5家、省级农民专业合作社示范社12家、国家级3家，农产品就地加工转化率达71%，村级电商服务点覆盖率达62%，永昌县电子商务进农村综合示范项目顺利通过商务部第三方绩效评估。

列支农村人居环境改善专项资金5620万元，全域无垃圾治理、村容村貌整治、清洁能源改造、“厕所革命”扎实推进，打造美丽乡村示范村6个，其中省级4个，整治“畅返不畅”农村公路195公里，改建农村集中居住点37个住宅1218套，改造农村危房328套，拆违拆旧拆废房屋1755套，改造土炕1万户。

——文旅融合发展实现新突破。航天科普基地“太空小镇”一期项目火星模拟生存基地建成投用，大型航天科普教育节目《挑战吧！太空》在优酷平台上线播出，中国青少年航天科普系列活动“太空C计划”启动实施，第六届金昌骊靬文化旅游节、第四届香草文化旅游节暨薰衣草之约集体婚礼、首届汽车越野拉力赛、金昌国际马拉松赛和全国残疾人乒乓球挑战赛成功举办，金昌知名度、美誉度大幅提升。金川区戈壁香草园、骊靬遗址保护开发等项目进展顺利，南坝滑雪场建成投运，金川区“十里花海”车窗景观带、永昌县车辘沟石林景区等乡村旅游基础设施不断完善，新建旅游厕所17座。积极融入“一部手机游甘肃”平台，智慧旅游“三个平台”不断完善，预计全年接待旅游人数493.56万人次、同比增长20%，实现旅游综合收入28.35亿元、增长26%。骊靬文化展示中心等项目进展顺利，金昌博物馆开馆投用，新建社区综合文化服务中心8个、24小时智能图书馆3个，改造提升“乡村舞台”30个。

——生态环境呈现新变化。按期完成祁连山生态环境、中央和省级环保督察反馈、党政主要领导干部自然资源资产离任审计等问题整改年度任务，“绿盾2018”专项行动扎实开展，祁连山金昌境内生态环境持续好转，污染防治攻坚战取得阶段性成效。“河长制”全面落实，“湖长制”积极推行，国防工业基地蓄洪生态工程、金川河道治理等项目有序推进，城市供水应急备用水源项目达到通水条件，地表水、地下水、饮用水水质优良率100%。全域国土绿化和防沙治沙行动扎实推进，重点生态林业年度工程全部完成，人工造林2.12万亩、封山育林6.5万亩、森林抚育1.8万亩。金川集团热电公司2&times;150MW机组超低排放改造、金昌发电公司一厂1号2号机组超低排放改造、永昌县污水处理厂提标改造、县城热源厂环保设施升级改造等项目建成投运，危废资源化处置、含镍钴废水处理系统升级改造等项目加快建设，万元工业增加值用水量下降5.01%，工业固废综合利用率达65.2%，主要污染物排放量控制在下达指标内，全年环境空气质量优良天数311天。

——改革开放有了新进步。政府机构改革全面启动，“放管服”改革扎实开展，“四办”改革成效明显，政务服务窗口进一步整合优化，市级一体化政务服务平台、市民服务热线平台建成投用，“双随机一公开”工作深入推进，“证照分离”改革全面推行，取消证明事项67项，企业开办时间压缩至5个工作日以内，梳理公布依申请类政务服务事项778项，政务服务事项全部实现“一网通办”，政务大厅服务事项全部实现“一窗受理”，15部政务服务热线实现“一号对外”，80%以上政务服务事项实现最多跑一次。税收征管体制改革全面完成，税费减免政策落实到位，累计为企业减税减负6.89亿元，预计非公经济增加值占GDP比重29.5%。事业单位和国有企业公务用车、国有企业“三供一业”、城管执法体制、农垦土地确权发证、供销、水价等改革稳步推进，农村产权交易中心加快建设，农村“三变”改革试点工作、农村集体资产清产核资通过省级验收。新型城镇综合改革试点加快推进，河西堡镇“区政合一”模式被国家发改委列为第一批国家新型城镇化综合试点典型经验。金川集团公司入选全国“国企改革双百行动”，金化集团公司改革重组稳步推进，国有资产监督不断强化，政府债务余额控制在限额之内。组团参加首届中国国际进口博览会，采购签约金额达90多亿元。积极支持金川集团公司、八冶建设集团公司开拓国际市场，扶持和鼓励非公外贸企业扩能改造，新增外贸企业18家，预计实现进出口贸易总额125亿元，同比增长40%。

——民生保障得到新提升。省上下达和市上确定的民生实事全面落实，城乡低保分别提高到674元/月、3720元/年，城乡特困供养标准分别提高到10510元/年、8067元/年，380户城市困难职工实现脱困解困，城镇新增就业15122人，登记失业率2.87%，永昌县被认定为省级农民工返乡创业示范县。教育现代化省级试验区全面建成，示范性综合实践基地、市三中新校区建成投用，龙门学校、永昌四小等项目进展顺利。全国健康城市试点稳步推进，公共卫生服务均等化水平不断提升，市第一人民医院迁建、八冶康颐养老一期等项目建成投用，市人民医院迁建、永昌中医院迁建项目基本建成。全国文明城市成果进一步巩固提升，国家卫生城市顺利通过复审，金昌市和金川集团公司分获2018全国质量魅力城市和质量标杆企业。严格落实“党政同责、一岗双责、齐抓共管、失职追责”制度，省安委会安全生产巡查反馈的281个问题整改完成266个，省政府对金川集团公司督查会诊反馈的125个问题督促完成整改115个。“平安金昌”建设扎实推进，“扫黑除恶”专项斗争取得阶段性成效，矛盾纠纷和信访积案有效化解，基层协商民主试点工作进展顺利，共建共治共享社会治理格局初步形成，人民群众获得感、幸福感、安全感不断增强。统计、人防、气象、档案、公积金、防震减灾等工作扎实推进，民族宗教、双拥共建、妇女儿童、侨务外事、慈善事业取得新进展。

——政府自身建设展现新风貌。认真落实全面从严治党主体责任，树牢“四个意识”，坚定“四个自信”，坚决做到“两个维护”，“两学一做”学习教育常态化制度化持续推进，政府系统政治建设和意识形态工作全面加强，党员干部对以习近平同志为核心的党中央政治认同、思想认同、理论认同和实践认同进一步增强。认真贯彻中央八项规定及其实施细则精神，全面落实国务院廉政工作会议精神，严格遵守廉洁自律准则和省政府“十不准”规定，切实履行党风廉政建设“一岗双责”，深入开展“转变作风改善发展环境建设年”和“深化放管服改革工作突破年”活动，扎实推进扶贫领域腐败和作风专项治理，国务院大督查、省委巡视和省纪委监委第三督查调查组反馈问题整改取得阶段性成效。法治政府建设全面加强，修订完善《政府工作规则》，出台实施政府任命工作人员宪法宣誓办法，政府系统学习党章、遵守党章、贯彻党章、维护党章和尊崇宪法、学习宪法、遵守宪法、维护宪法、运用宪法蔚然成风，科学化、民主化、法制化决策水平进一步提高。自觉接受人大依法监督、政协民主监督和社会各界监督，办理人大代表意见建议97件、政协提案191件，办复率达100%。

各位代表，过去的一年，我市经济社会取得了一些重大发展成果，主要经济指标逆势向好，实现止滑回升、稳定增长，增幅在全省排名靠前；脱贫攻坚取得决定性成果，插花型贫困县永昌县如期脱贫摘帽；地企深度融合发展进入新境界，在拓宽拉长产业链、互帮互助解难题等方面迈出了新步伐，合作共赢发展取得了新成效；企业投资项目审批时限压缩至100天以内，完成时间和压缩时限均在国务院提出2019年6月份压缩至120天的目标以内；“太空小镇”脱颖而出，“太空挑战”成为金昌新名片；“放管服”改革成果丰硕，市、县区政务服务事项网上可办率均达到100%，均位列全省第一名，等等。这些发展成果是我们认真贯彻新思想、全面落实新理念、阔步迈入新时代的生动实践，是我们以实际行动践行坚决做到“两个维护”、统筹推进“五位一体”总体布局、协调推进“四个全面”战略布局取得的工作成效，是我们深入落实习近平总书记视察甘肃重要讲话和“八个着力”重要指示精神的担当体现，也是我们纪念改革开放40周年的最好献礼！

各位代表，成绩来之不易，这是省委、省政府和市委科学决策、正确领导的结果，是市人大、市政协监督支持、共识共为的结果，是地企、军地密切配合、通力协作的结果，是全市人民艰苦奋斗、和谐进取的结果。在此，我代表市人民政府，向过去一年所有关心、支持和参与金昌改革发展的各族各界人士，表示衷心的感谢！

在总结成绩的同时，我们也清醒的认识到，经济社会发展和政府工作还存在不少困难和问题：一是发展质量和效益有待提高，经济运行中的痛点、难点、堵点还比较多，企稳向好的基础还不牢固，新旧动能转换动力不足；二是城乡二元结构矛盾依然较为突出，“空心村”“空巢村”问题日益显现；三是安全生产形势依然严峻，一些社会事业和公共服务还比较滞后，保障和改善民生与群众期盼还有一定差距；四是全面深化改革的力度、进度不平衡，金融风险、企业风险、政府债务风险不容忽视；五是生态环境建设任务繁重，环境质量持续改善压力大；六是营商环境还需进一步改善，“放管服”改革力度还需加大；七是巡视、督察、审计反馈的一些问题还没有完全整改到位，还需持续用力、久久为功；八是政府系统一些干部职工还存在“不作为、慢作为、怕担责”的现象，作风建设和廉政建设还需进一步加强，等等。对于这些问题，我们一定高度重视，勇于担当，认真解决，决不辜负全市人民的期盼！

二、2019年主要工作

2019年，是新中国成立70周年，是全面建成小康社会、实现第一个百年奋斗目标的关键之年，也是推动金昌高质量发展的重要一年，做好政府各项工作至关重要。政府工作的基本要求是：坚持以习近平新时代中国特色社会主义思想为指导，全面贯彻党的十九大和十九届二中、三中全会精神，深入贯彻落实习近平总书记视察甘肃重要讲话和“八个着力”重要指示精神，围绕统筹推进“五位一体”总体布局和协调推进“四个全面”战略布局，坚持稳中求进工作总基调，坚持新发展理念，坚持推动高质量发展，坚持以供给侧结构性改革为主线，坚持深化市场化改革、扩大高水平开放，认真贯彻落实党中央和省市委各项决策部署，全面落实市委八届六次全会确定的“十个坚持”和市委八届七次全会明确的目标任务，统筹推进稳增长、促改革、调结构、惠民生、防风险工作，保持经济持续健康发展和社会大局稳定，为全面建成小康社会收官打下决定性基础，以优异成绩庆祝中华人民共和国成立70周年。

结合“十三五”规划目标，提出2019年全市经济社会发展的主要预期目标是：地区生产总值增长6%左右，人均地区生产总值增长6%，全员劳动生产率达到9.8万元/人，制造业增加值占生产总值比重达到14%，规模以上工业增加值增长6%左右，固定资产投资增长6%左右，一般公共预算收入增长6%左右，社会消费品零售总额增长8%左右，进出口贸易总额增长5%左右，城镇和农村居民人均可支配收入分别增长7%和8%左右，居民消费价格指数涨幅控制在3%以内，城镇登记失业率控制在4%以内，单位GDP能耗控制在省上下达的指标以内。

为实现上述目标，我们必须坚决贯彻“八字方针”，下大力气在巩固“三去一降一补”成果、增强微观主体活力、提升产业链水平、畅通经济循环方面取得更大突破，重点抓好以下八个方面的工作：

（一）高质量实施“工业强市”战略。围绕产业集群有规模、产业链条有延伸、循环利用有突破、质量品牌有提升、环境容量有空间，深入推进地企融合发展，着力打造“工业强市”升级版，加快构建现代化工业体系。

提升科技创新能力。坚持把创新作为引领高质量发展的第一动力，深入实施创新驱动战略，加快构建以科技创新为核心，多领域互动、多要素联动的综合创新生态体系。围绕建设省级创新型城市，积极支持规上企业建立技术研发中心、柔性产学研用联盟和科技创新团队，构建开放、协同、高效的共性技术研发平台，健全需求为导向、企业为主体的产学研一体化创新机制，增强技术创新能力。深入开展省级科技成果转移转化示范区创建，建立科技资源开放共享网络平台，着力开发引进转化新技术、新成果，申请专利880件以上，万人有效发明专利拥有量达到7.8件以上。积极支持金川集团公司重大科研基础设施、大型科研仪器、专利基础信息资源等向社会开放，实现科技资源的开放整合与高效利用，打造产业链配套的创新型产业集群。大力弘扬企业家精神和“工匠精神”，引进培养专业型、实用型、复合型人才，推行企业科技特派员制度，提升孵化机构和众创空间服务水平，形成线上线下结合、产学研用协同、大中小企业融合的创新创业格局，打造“双创”升级版，培育高新技术企业6家、科技型中小微企业10家，研究与实验发展经费支出与地区生产总值比例达到4%。

培育战略性新兴产业。认真组织实施先进制造、军民融合等生态产业专项行动计划，加快国家镍钴新材料高新技术产业基地建设，支持金川集团公司实施企业绿色化改造工程、开展全省新能源汽车动力蓄电池回收利用试点，推进镍网、电池、表面处理、粉末冶金、电工材料、铜镍合金加工材、军民融合等七大产业链建设，不断提升产业链水平，培育和发展新的产业集群。积极顺应5G发展趋势，加快发展数字经济，推动互联网、大数据、人工智能、区块链和传统经济、实体经济相融合，鼓励有实力的企业主动引进新技术，发展无人工厂、3D打印、软件信息等新业态；主动融入全省丝绸之路“两港”建设，实施工业互联网平台培育工程，开展企业上云计划，加快推进“紫金云”大数据项目，打造全国重要的云计算产业聚集区和数据灾备库、离岸数据中心。争取国家国防科技工业军民融合产业投资基金，开发高温合金用镍、航空航天用变形高温合金加工材、军工吸波屏蔽材料用羰基铁粉、高温合金母合金等产品，实施2000吨新能源电池覆钴正极材料等项目，打造西部地区最大的电工、有色金属粉体、中国航空航天军工镍钴金属材料研发生产基地，推动有色金属新材料产业链向中高端发展。

升级改造传统优势产业。大力实施清洁生产生态产业专项行动计划，全面提高传统产业发展水平。着力推进危化品企业搬迁工作，支持金昌奔马化工、金昌裕隆达气体、永昌县科瑞化工、甘肃麦格理钾肥等4家企业异地迁建和瓮福化工就地改造，推动无机化工向有机化工和精细化工发展，锻造基础化工发展新优势。着力加快信息化和工业化融合发展，大力实施数据信息生态产业专项行动计划，推进金泥集团公司电石炉生产智能化改造等项目，支持金川集团公司企业资源计划系统一期、资产管理系统开发应用等项目建设，鼓励和引导重点制造企业实施流程再造示范试点，加快关键工序智能化、关键岗位机器人替代，锻造信息化和工业化融合发展新格局。着力实施瓮福化工10万吨建筑石膏粉、双虎6万吨岩棉二期等项目，锻造绿色建材发展新业态。

强化工业企业运行调度。按照“一业一策、一企一策、一事一议”的原则，全面落实降低一般工商业用电价格政策，积极做好煤电油气运保障工作，有效降低企业要素成本，新增规上工业企业6户以上。围绕建成项目，推动3万吨动力锂离子电池用三元前驱体、3.5万吨硫酸镍扩能改造、5000吨甲基磺酰氯等项目全面投产，充分释放产能；围绕存量资产，积极支持金川集团公司与新希望集团公司氯碱化工循环产业链合作框架协议落实，推动烧碱、PVC、硫酸钾等存量闲置资产尽快复产、发挥效益；围绕生产企业，推动金泥40万吨电石、鑫华焦化2号炉、兰石集团循环硫化床、丰盛科技20万吨合成氨等项目和氯碱化工产业链达标达产，带动上下游关联企业扩大生产负荷，实现全产业链循环高效运转。

（二）高质量实施乡村振兴战略。把实施乡村振兴战略作为新时代“三农”工作的总抓手，坚持农业农村优先发展，加快推进农业农村现代化，努力让农业成为有奔头的产业，让农民成为有吸引力的职业，让农村成为安居乐业的美丽家园。

促进农业全面升级。切实抓好粮食生产，推动藏粮于地、藏粮于技落实落地。着力发展现代丝路寒旱农业，认真实施循环农业生态产业专项行动计划，加快发展戈壁农业，构建质量品牌、市场对接、冷链储运服务、农产品加工、风险担保等现代农业“五大体系”，增加优质绿色农产品供给。发展以娃娃菜、胡萝卜、食用菌、西芹等为主的高原夏菜产业，抓好金禹川蔬菜产业化基地、金海彧优质蔬菜产销一体化、尾菜处理和综合利用等项目，落实蔬菜种植面积28万亩以上，新改建食用菌棚5000平方米，打造精品蔬菜标准园10个，培育2万亩以上规模的蔬菜重点乡镇5个。发展以优质牛羊肉、紫花苜蓿为主的草食畜牧产业，人工饲草面积稳定在20万亩，新改建标准化养殖场8个，开工建设瑞嘉万头奶牛、居佳生态万只肉羊、红光万只奶绵羊生态牧场一期等项目，打造优质肉羊养殖基地和奶源生产基地，加快畜牧业转型升级与绿色发展步伐。发展以薰衣草、万寿菊、油菜花为主的香草花卉产业，重点实施金川区戈壁香草园等项目，进一步优化农业产业和产品结构。推进农业质量品牌体系建设，加快甘肃省藜麦产品质量监督检验中心建设，争创国家农产品质量安全市，引进推广应用农业新技术20项、新品种60个，创建市级以上农业科技示范园区4家、农业科技示范点30个。

促进农村全面进步。坚持规划先行、示范引导，因地制宜、分类指导，统筹兼顾、有序推进，打造各具特色的现代版“富春山居图”。切实加强基础设施建设，着力开展农村宅前硬化亮化和房后绿化美化，大力发展高效节水农业，新修“四好农村路”100公里，整治“畅返不畅”农村公路140公里，发展高标准农田12万亩、高效节水灌溉4.7万亩。切实加强村容村貌整治，深入学习浙江“千万工程”经验，推进农村人居环境整治三年行动、全域无垃圾专项治理和村庄清洁行动，巩固提升“全国厕所革命优秀城市”成果，加快农村污水垃圾收集处理设施建设，大力改造农宅风貌、拆除破旧房屋，新改建公建民住居住点16个，完成3802户农宅风貌改造，拆除破旧房屋2855户。切实加强清洁能源改造，重点围绕祁连山浅山区清洁能源替代，加大财政支持力度，着力推进煤灶改电改气、清洁取暖改造工作，完成土炕改造8800户。切实加强文明乡风培育，加快建设新时代文明实践中心，发挥群众自治组织和村规民约的教化约束作用，倡导婚事新办、丧事简办、他事俭办、文明理事新风尚，培育文明乡风、良好家风、淳朴民风，提升乡村社会文明程度。

促进农民全面发展。打赢打好脱贫攻坚战，坚持扶贫与扶志扶智相结合，实施“精神扶贫”工程，激发贫困群众参与脱贫攻坚的内生动力；坚持脱贫不脱政策、脱贫不脱责任，统筹用好扶贫专项资金和特色产业发展工程贷款，切实巩固脱贫攻坚成果。把增加农民收入和发展壮大村级集体经济有机结合起来，在每个行政村建立金融综合服务室，有力有序发展新型经营主体，推进农民合作社、家庭农场等新型农业经营主体规范化建设和示范创建工作，发展市级以上示范性农民合作社15家、家庭农场10家，培育壮大生产、加工、销售等环节龙头企业2家以上，辐射带动农民务工收入持续增加。抓实抓好农民技能培训，依托甘肃有色冶金职业技术学院、金川公司技校、八冶公司技校等各类培训基地优势，着力开展电焊工、建筑工、机械工和家政服务、现代农业种养等技术培训，重点培养工业企业产业工人、现代农业技术能手、服务产业行家里手，建成扶贫车间20个，培训新型职业农民3万人次，农民转移就业7万人以上，提升农民自我发展、增收致富能力。

（三）高质量推动第三产业发展。坚持把发展壮大第三产业作为转方式、调结构、稳就业的关键点和突破口，深入推动产业融合多元发展，促进现代服务业优化升级，不断增强高质量发展竞争力和创造力。

突出旅游富民乐民。按照全域旅游发展理念，大力实施文化旅游生态产业专项行动计划，围绕叫响“八大特色旅游名片”，重点加快太空小镇火星体验基地、航天员地外生存训练基地及道路工程等项目建设，打造全国一流航天科普旅游目的地。继续办好国际青少年生存训练营、金昌国际马拉松等精品赛事活动，认真落实与省公航旅集团达成的战略合作协议，深度开发婚庆度假、研学体验、户外运动、自驾露营等特色旅游产品和个性化旅游项目，实施漠北文化旅游、双湾镇陈家沟关帝庙民俗文化体验区等项目，支持鼓励民营资本投入文化旅游产业，发展壮大文化旅游市场主体，培育乡村旅游示范村4个，形成“旅游+”新业态全方位发展格局，全年接待旅游人数同比增长16%，旅游综合收入增长18%，使旅游产业真正成为富民乐民、创业兴业的优势产业。

突出文化育民惠民。坚持把培育和弘扬社会主义核心价值观作为凝魂聚气、强基固本的基础工程，加强核心价值观景观工程建设，强化文化遗产传承保护利用，加快构建现代公共文化服务体系，推进公共文化服务标准化均等化。积极创建“志愿之城”，实施“美丽乡村·文明家园”行动，深化群众性精神文明创建活动，组织开展文明礼仪、文明交通、文明旅游、文明餐桌、文明劝导、文明上网等行动，进一步巩固全国文明城市成果。充分发挥“乡村舞台”等文化科普载体作用，大力开展“红色文艺轻骑兵”文化惠民进万家活动，继续组织好公益性文艺巡演、农村公益电影放映和送戏曲、书画、图书进乡村进校园等文化惠民工程，抓好基层数字图书馆和智慧广电、市档案服务中心、县级融媒体中心建设，设立“金昌文艺奖”，鼓励文艺创作，讲好金昌故事，传播金昌声音。

突出消费利民便民。认真实施通道物流生态产业专项行动计划，做大做强融资租赁、家政养老、信息服务等现代服务业，改造提升交通运输、批发零售、餐饮住宿等传统服务业。按照“城市有物流配送中心、乡村有电商服务平台、产地有产品批发市场、社区有综合服务网点”的要求，加快农产品产地批发市场、便民市场和农业电商公共服务等项目建设，推进特色农产品冷链物流建设，发展集零售、餐饮、娱乐于一体的多功能乡镇商贸中心，进一步完善电商三级服务体系，破解工业品下乡、农产品进城“最后一公里”问题，电子商务交易额达2.6亿元以上。顺应城乡居民需求新变化，培育网络消费、定制消费、体验消费、智能消费、时尚消费等消费新热点，鼓励引导文化、体育、养老、健身等领域消费，支持自驾游、旅居等大众消费，推动消费升级提质扩容，努力让老百姓吃得放心、穿得称心、用得舒心。

（四）高质量推动项目建设。坚持把项目作为扩大有效投资的主要抓手和拉动经济增长的重要力量，旗帜鲜明抓项目建设，以项目建设推动经济社会高质量发展。

抓好项目谋划。立足资源禀赋，对标高质量发展要求，着眼优势产业发展壮大，聚焦“十大生态产业”，紧盯九大短板领域，围绕可准入、可落地、可申报、可持续和补短板、延链条、保脱贫、促转型，正确把握国家宏观政策、结构性政策、社会政策取向，找准国家政策、产业布局和资金投向的对接点，认真谋划一批“含金量”“含新量”“含绿量”高的重大项目，切实增强项目谋划的科学性、前瞻性和有效性。抢抓“一带一路”、新时代西部大开发、基础设施领域补短板、国际陆海贸易新通道、生态环境修复、乡村振兴、军民融合等政策机遇，按照对口争取、密切跟踪、精准对接的要求，加大政策窗口期跑项目、争资金、增投资力度，重点争取省上出台镍铜钴高质量发展支持政策，力争有更多高质量项目进入国家和省上规划盘子、获得支持，确保争取份额最大化、利益最大化。

抓好项目建设。坚持实行重大项目包抓、项目清单管理及中央预算内投资重大项目库月调度等机制，实施新续建项目257个，完成投资122亿元，重点项目开工率达到90%以上。支持二矿区深部开采、三元前驱体正极材料、废旧电池智能化处理、铬锆铜新材料、贵金属一二次资源回收利用、八冶康颐养老二期等项目建设，推动20万吨联碱、3.5万吨电镀用高端镍盐等项目建成投产，加快5万吨动力电池级硫酸镍、G570金昌（下四分）至永昌及省道S212线红沙岗至下四分一级公路改扩建工程二期等项目建设，积极配合做好兰州至张掖铁路三四线武威至张掖段项目前期工作，增强高质量发展后劲。

抓好项目招商。坚持把招商引资作为推动高质量发展的强力引擎，围绕金川集团公司下游产品开发及产业链延伸，以产业链、供应链、创新链深度融合为方向，突出专业招商、精准招商、主题招商、地企联合招商，用心用情用力抓招商，聚焦聚神聚力抓引资，全力以赴推动招商工作高质量发展。切实加强金昌经济技术开发区、河西堡化工循环经济产业园区、永昌县工业园区、金川区中小企业承载园基础设施建设，加快金耀控股5万吨电子铜箔材料、国科桑田液态阳光产业园等招商项目落地实施，进一步延伸有色金属深加工、PVC深加工、硫磷化工、资源综合利用、生产性服务业等产业链条。建立招商项目快速落地综合协调机制，认真落实招商引资优惠政策，持续抓好“兰洽会”签约项目跟踪服务，促进更多签约项目尽快落地建设。

（五）高质量推动生态文明建设。牢固树立和践行“绿水青山就是金山银山”的理念，坚持把绿色作为推动高质量发展的普遍形态，自觉扛起生态文明建设政治责任，严守“三条红线”，坚决打赢打好污染防治攻坚战，让金昌天更蓝、山更绿、水更清、环境更优美。

着力加强生态文明制度体系建设。实行最严格的生态环境保护制度，全面落实环境保护“党政同责、一岗双责”“一票否决”、党政主要领导干部自然资源资产离任审计等制度，严格执行生态环境损害赔偿制度和损害责任终身追究制，进一步健全落实资源有偿使用和生态补偿机制，加快建立生态文明差别化考核制度和环保责任常态化监管机制，深入开展“绿盾2019”自然保护区专项行动和“大棚房”问题专项清理整治，依法严厉打击违法排污和破坏生态环境行为。积极倡导勤俭节约、绿色低碳、文明健康的生产方式、生活方式和消费方式，推动生态文明建设人人参与、生态文明成果人人共享。

着力加强生态工程建设。以防沙治沙为重点，统筹抓好祁连山林地保护与建设、退耕还林还草、湿地保护与修复、“三北”防护林、国家级公益林管护等重大生态工程，完成金川河道治理、引硫济金工程引水隧洞出口维修加固及环保治理等任务。稳步推进大规模国土绿化行动，落实防沙治沙三年行动计划，配合做好祁连山国家公园体制试点工作，切实加强祁连山水源涵养林建设和芨芨泉省级自然保护区生态环境保护，全面管护沙区公益林，完成人工造林4.28万亩、封山育林2.5万亩、森林抚育2.5万亩，为高质量发展提供生态保障。加强水资源节约和利用，开展全民节水行动，实行水资源总量和定额管理，严控地下水超采，全面推进节水型社会建设。

着力加强环境污染整治。以祁连山生态环境和中央、省级环保督察反馈问题整改为重点，深入落实“气十条”，认真开展“蓝天”行动，严格实施“四控一管”，强化大气污染防治，推进生态环境监测网络建设，建立“天空地一体化”动态生态监测网络，实施春天化工2&times;30MW三废综合利用发电等项目，强化秸秆焚烧监管和柴油货车污染治理，主要污染物排放总量控制在省上下达指标之内。深入落实“水十条”，认真开展“碧水”行动，严格落实“河长制”“湖长制”，严守水资源开发利用控制、用水效率控制和水功能区限制纳污“三条红线”，推进水污染治理、水生态修复和水资源保护“三水共治”，加快含镍钴废水处理系统升级改造、金川区农村污水处理等项目建设，逐步实现污水管网全覆盖、全收集、全处理。深入落实“土十条”，认真开展“净土”行动，强化农村面源污染治理，开展土壤污染源调查及工业危险废物专项整治行动，加强矿山地质环境保护与治理恢复，加快城市第二生活垃圾处理场、危废资源化处置、农村垃圾处理等项目建设，建立农膜回收利用、尾菜处理、秸秆资源化利用等长效机制，废旧农膜利用率达到81%以上，尾菜处理率达到46%以上，秸秆综合利用率达到84%以上。

（六）高质量推动城市发展。按照建设幸福美丽金昌的发展定位，统筹规划、建设、管理三大环节，优化生产、生活、生态三大布局，着力塑造有颜值、有魅力、有活力的城市新形象，形成高质量发展的重要推动力。

塑造特色魅力金昌。尊重城市发展规律，坚持“五个统筹”，贯彻“实用、经济、绿色、美观”的方针，着力转变城市发展方式、塑造城市特色风貌，提升城市规划水平。坚持以提升综合创新力、城市竞争力、可持续发展能力和镍都独特魅力为重点，编制完成《金昌市城市总体规划2018—2035》《金昌市城市双修专项规划》和中心城区部分小区控制性详细规划，完善县城和乡镇发展规划及美丽乡村、村庄建设规划，绘制统筹发展“一张蓝图”，明确城市未来的远景目标、功能定位、发展战略、行动计划和阶段性任务，进一步优化城镇空间格局和产业布局，更多体现金昌地域特色和新时代特征。

打造智慧信息金昌。认真落实智慧城市发展总体规划，按照“能整合的整合、应共享的共享、应开放的开放”的基本原则，充分利用现有信息化基础资源，加快推动“互联网+”、大数据、人工智能和物联网等新一代信息技术与经济社会运行全要素深度融合，实现数字资源共建共享、集约使用，逐步构建新型智慧城市体系。积极推进文化旅游、产业发展、生态环保、民生服务、城市管理、电子政务六大关键领域智慧应用创新，提升城市信息化、智能化管理水平，逐步将金昌建设成为区域融合的智慧枢纽、产业创新的智慧基地和谐宜居的智慧典范。

建造宜居宜业金昌。巩固国家卫生城市、国家园林城市成果，创建全国质量强市示范城市，提升城市经济品质、人文品质、生态品质、生活品质，增强城镇集聚能力，常住人口城镇化率达到72%以上。进一步深化城市管理执法体制改革，持续开展市容环境综合治理，强化居民小区物业管理，全面推行垃圾分类制度，实施立面整治、街净巷洁、路平桥安、城绿水清、灯明景靓、治乱拆违等工程，重点疏导规范犬类散养、占道经营、露天烧烤等无序乱象，整治街巷烧纸、乱搭乱建、乱停乱放等违法行为，提高城市精细化管理水平。完成泰安路、建设路与公园路交叉路口等城市道路改造，实施智慧绿色交通、城市公共交通综合建设、电动汽车充电基础设施、电网改扩建、中水回用管道和永昌县热电联供、天然气综合利用等项目，新增新能源公交比重达到25%，逐步解决“逢雨必涝”“马路拉链”“交通拥堵”等突出问题。

（七）高质量推动改革开放。坚持改革不忘初心，加大重点领域和关键环节改革力度，增强改革方案的针对性、可行性，提高改革举措的执行力、落实力，为高质量发展增活力、添动力。

抓好重点领域改革。稳妥有序做好政府机构改革工作，优化政府机构设置和职能配置，加强乡镇政府服务能力建设，推进河西堡镇行政管理体制改革试点工作，形成职责明确、依法行政的政府治理体系，增强政府公信力和执行力。深化“放管服”改革，加快推进政务服务“一网、一门、一次”改革，完善“双随机一公开”监管机制，积极开展“证照分离”改革，加快推行“模拟审批”“容缺审批”“多规合一”“多图联审”“联合验收”“区域评估”等工作机制，深化“互联网+政务服务”体系建设，打通信息孤岛，努力让“数据多跑路、群众少跑腿”。稳步推进农村“三变”、农村集体产权制度和水利工程水价改革，加快建设农村产权交易市场，强化农村“三资”平台监管，激活农村资源要素配置。加快国资国企改革，组建国有资本投资运营公司，推进市直部门管理企业改制脱钩集中统一监管，实现从管企业向管资本转变；稳妥有序开展国有企业剥离办社会职能工作，支持金川集团公司整体上市，引导金化集团公司与战略投资者开展资本股权合作，实现资产重组。深化投融资体制改革，强化投融资平台监管，稳步推进交通领域“四种投融资模式”落地实施，充分利用政府债券、PPP、产业基金等模式筹集项目建设资金，拓宽基础设施建设项目资本金来源渠道。坚决打赢打好防范化解重大风险攻坚战，建立健全政府债务风险防控机制，全面实施预算绩效管理，着力遏制隐形债务增量，妥善化解债务存量，做到坚定、可控、有序、适度。

大力发展民营经济。坚持权利平等、机会平等、规则平等，建立健全企业参与营商环境政策制定、以企业为主评估营商环境等管用机制和优秀企业家表彰奖励办法，发挥企业和企业家主观能动性，积极发展混合所有制经济，实现“国进民升、互促共生”。建立规上工业企业和重大项目服务专员工作机制，为企业从设立到成长、从发展到壮大的全生命周期提供 “保姆式”“贴近式”“零距离”专员服务，助推民营经济高质量发展。坚决砍掉不合法不合理的涉企收费事项，切实降低各类营商成本，保护民营企业家人身安全和财产安全，让企业家专心创业、放心投资、安心经营。围绕新材料、新能源、装备制造、精深加工、公共服务等领域，加快推进金昌民营企业产业园区建设，扶持发展一批科技含量高、经济效益好、带动作用强的中小型企业。积极推动宇恒镍网等民营企业股份制改造和上市辅导，加强金融机构与重点企业、重点项目的对接合作，提高土地、房产、林权、知识产权等抵押率，引导金融机构强化绩效考核，提高金融体系服务实体经济能力，切实解决民营企业融资难、融资贵问题，为中小企业发放“助保贷”“互助贷”余额3亿元，非公经济增加值占GDP比重达到31%左右。

扩大对外开放合作。坚持把开放作为推动高质量发展的必由之路，积极参与“一带一路”建设，围绕抢占“文化、枢纽、技术、信息、生态”五大制高点，借助丝绸之路国际文化博览会、循环经济示范区等国家级战略平台，积极融入国际陆海贸易新通道建设，推行国际贸易“单一窗口”一站式服务，提高贸易便利化水平。支持金川集团公司印尼红土镍矿和八冶建设集团公司玻利维亚穆通综合钢厂选矿工程等重点项目建设，鼓励宇恒镍网、御宝泽等外贸企业扩能增量改造，拓展特色产品外销市场。

（八）高质量提高人民群众生活品质。紧紧围绕解决好人民群众普遍关心的突出问题，持续推进“打造共建共治共享社会治理格局”试点工作，下功夫办好顺民意得民心惠民生的实事好事，不断增强人民群众获得感、幸福感、安全感。

提高社会共建水平。把稳就业摆在突出位置，统筹做好高校毕业生、城镇就业困难人员、退役军人、返乡农民工、进城农民等就业创业工作，保障农民工劳动报酬权益，新增城镇就业1万人以上，完成就业、创业等各类培训8500人次。多措并举促进教育质量提升和均衡发展，进一步深化教育体制机制改革，提升校长办学治校能力，加大教师分级分层培训力度，推行集团化办学和联盟办学，着力解决中小学生课外负担重、“择校热”、大班额等突出问题，完成龙门学校、永昌四小等项目，新建幼儿园2所。积极开展国家健康城市试点工作，认真实施中医药生态产业专项行动计划，深化医药卫生体制改革，健全现代医院管理制度，加强基层医疗卫生服务体系和全科医生队伍建设。

提高社会共治水平。坚持发展新时代“枫桥经验”，推进社会治理体系和治理能力现代化。加快建设社会治理网格化管理工作新模式，探索实行“街（巷）长”负责制，创新村（居）民议事形式，健全完善村（社区）依法治理架构。深化“扫黑除恶”专项斗争，加快推进“雪亮工程”和综治中心规范化建设，构建现代化、立体化、信息化、智能化治安防控新体系。进一步加强信访法治化建设，积极预防和化解社会矛盾，加快建立健全社会矛盾纠纷多元化解工作格局。深刻汲取“7·12”“10·5”安全生产事故教训，进一步树牢安全红线意识，创建安全发展示范城市，推进龙首新区消防站建设，强化灾害监测预警、风险防控和应急处置工作，坚决遏制重特大事故发生。扎实开展打击食品药品农资环境烟草领域违法犯罪专项行动，全域创建食品安全城市，切实维护人民群众生命财产安全和身体健康。不断强化民族宗教事务依法管理，促进各民族相互交往交流交融，保持宗教领域和谐稳定。

提高社会共享水平。按照“兜底线、织密网、建机制”的方针，构建“覆盖全民、城乡统筹、权责清晰、保障适度”的可持续多层次保障体系，不断提升保障服务能力和社保待遇水平。准确把握住房的居住属性，积极争取地方政府棚改专项债券配额，规范住房租赁市场，抑制房地产泡沫，满足新市民住房需求。完善城镇养老、医疗等社会保险制度和城乡最低生活保障制度，统筹规划建设城乡养老服务设施，探索“互联网+养老”、医养结合等服务新模式，鼓励社会力量参与兴办养老机构。认真落实全民健身国家战略，做好甘肃省第四届中学生运动会承办工作。在全省率先全面推行第三代智能化残疾证试点工作，支持社会福利和慈善事业，提高特殊困难群体帮扶精准度和有效性。

各位代表，人民群众对美好生活的向往就是我们的奋斗目标。2019年，市政府在落实好省政府确定的利民惠民实事的同时，将集中力量办好10件民生实事：1。实施农村饮水安全巩固提升工程，确保1949户供水安全；2。做好城镇棚户区改造工作，新开工项目35个31271套（户），建成项目5个1545套（户）；3。新建8个社区综合文化服务中心，改造提升25个行政村综合文化服务中心（乡村舞台）；4。抓好体育惠民工程建设，新建4个乡镇（社区）体育健身中心；5。认真落实厕所革命新三年行动计划（2018-2020年），积极推进农村改厕工作，新改建农村户用卫生厕所9420座，建制村公共卫生厕所普及率达到100%；6。对城镇常住人口高危人群免费开展肺癌、大肠癌、上消化道癌、乳腺癌和肝癌“五癌”筛查工作；7。新建或改造提升1个农产品产地批发市场，改造建设2个便民市场；8。全面提升城乡社区养老服务水平，新建城市社区日间照料中心2所、农村互助老人幸福院4个；9。全面实施残疾儿童康复救助项目，完成110名残疾儿童康复救助；10。扎实推进城市困难职工解困脱困工作，率先在全省实现建档立卡困难职工全部解困脱困。

三、全面加强政府自身建设

新时代呼唤新担当，新征程需要新作为。我们将牢记宗旨，锐意进取，严格落实全面从严治党要求，不断加强自身建设，深入推进职能转变，加快建设顺应人民群众新期待、适应实践发展新要求的新时代人民满意政府。

（一）坚持从严治政。坚持以习近平新时代中国特色社会主义思想为指引，不断加强政治建设，持续推进“两学一做”学习教育常态化制度化，扎实开展“不忘初心、牢记使命”主题教育，树牢“四个意识”，坚定“四个自信”，坚决做到“两个维护”，始终在政治立场、政治方向、政治原则、政治道路上同以习近平同志为核心的党中央保持高度一致，推动中央方针政策、省委省政府及市委工作部署落地生根。全面彻底整改巡视巡察、国务院大督查反馈问题。严格落实意识形态工作责任制，坚决做到守土有责、守土负责、守土尽责。

（二）坚持依法行政。严格遵守宪法法律，加快建设法治政府，坚持依法治市、依法执政、依法行政，深入实施“七五”普法规划，共同推进法治金昌、法治政府、法治社会一体建设。坚决服从市委领导，自觉接受人大法律监督、工作监督和政协民主监督，高质量办理人大代表意见建议和政协委员提案。坚持依法统计，坚决杜绝统计造假、弄虚作假和虚报瞒报等违纪违法行为，切实提高统计数据质量。对涉及重大公共利益、群众切身利益的事项，主动充分听取人民群众意见建议，积极推进决策公开、执行公开、管理公开、服务公开、结果公开。

（三）坚持为民勤政。坚持把勤政作为各级政府及其工作人员应尽的本分，以勇于担当、舍我其谁的精神状态，以“昼无为、夜难寐”的责任感和紧迫感，严格落实省政府“十不准”规定，抓铁有痕、踏石留印，进一步提升执行力和落实力。对贯彻执行中央、省市决策部署不力，未能完成经济社会发展约束性指标和硬任务的，该约谈的约谈，该通报的通报，决不迁就；对不尽职、不作为、不担当、假作为的，严肃批评、限期改正，公开曝光，决不听之任之；对失职渎职造成严重后果的，坚决按照党纪国法处理，决不姑息，切实增强政府工作人员的政治担当、历史担当、责任担当。

（四）坚持廉洁从政。严格落实全面从严治党主体责任和“一岗双责”，健全完善行政权力监督制约机制，从严查处侵害群众利益行为，保持惩治腐败“零容忍”高压态势。严格贯彻落实中央八项规定及其实施细则精神，持续深入开展“转变作风改善发展环境建设年”活动，驰而不息纠“四风”、树新风，特别要力戒形式主义、官僚主义。全面推进党政主要领导干部自然资源资产离任审计，实现对公共资金、国有资产、国有资源和领导干部履行经济责任审计全覆盖。严格执行廉洁准则和纪律处分条例，强化监督执纪问责，加强巡视巡察和审计结果运用，畅通社会监督、舆论监督渠道，让权力在阳光下运行，着力营造激浊扬清、干事创业的良好政治生态。

各位代表！幸福生活是奋斗出来的，美丽家园是双手创造的。让我们更加紧密地团结在以习近平同志为核心的党中央周围，在省委、省政府和市委的坚强领导下，在市人大、市政协的监督支持下，紧紧依靠全市人民，勇做新时代的奋斗者、建设者、实干者，艰苦奋斗、和谐进取，为推动金昌高质量发展、建设幸福美丽金昌努力奋斗！
